# Consequences of Purtscher-Like Retinopathy in a Patient With Systemic Lupus Erythematosus: A Case Report

**DOI:** 10.7759/cureus.47837

**Published:** 2023-10-27

**Authors:** Estefania Ramirez Marquez, Israel J Mendez Bermudez, Noraliz Garcia, Armando L Oliver

**Affiliations:** 1 Ophthalmology, School of Medicine, University of Puerto Rico, Medical Sciences Campus, San Juan, USA; 2 Ophthalmology, University of Puerto Rico, Medical Sciences Campus, San Juan, USA

**Keywords:** ophthalmology, pan-retinal photocoagulation laser, systemic lupus erythematosus, purtscher-like retinopathy, purtscher retinopathy

## Abstract

We report on a case of Purtscher-like retinopathy (PLR) secondary to systemic lupus erythematosus, which caused retinal ischemia, retinal neovascularization, vitreous hemorrhage, and, ultimately, a combined tractional and rhegmatogenous retinal detachment. A 23-year-old male presented with decreased visual acuity in the left eye (OS). He had a recent history of systemic lupus erythematosus and was being treated with systemic corticosteroids. At presentation, his visual acuity was counting fingers in the OS; a fundus examination of the OS revealed the presence of macular edema associated with flame hemorrhages, diffuse cotton-wool spots surrounding the macula, and vascular sheathing with the retina attached, all of which were consistent with PLR. Five months later, his fundus examination (OS) showed severe retinal ischemia and active neovascularization. The patient was scheduled for pan-retinal photocoagulation (PRP) laser therapy, but he was lost to follow-up. Subsequently, he returned two months later with progressive damage and was treated with PRP in the OS. An additional two months after PRP treatment, an examination revealed combined tractional and rhegmatogenous retinal detachment. Ultimately, the patient required a pars plana vitrectomy.

## Introduction

Purtscher retinopathy is thought to be vaso-occlusive retinopathy associated with painless diminished visual acuity synchronous with or up to two days after trauma [1-3]. It is characterized by Purtscher flecken, cotton-wool spots, and retinal hemorrhages [1-6]. Purtscher flecken is commonly described as retinal whitening that does not affect the retina directly beside the arterioles [1]. In the absence of a history of trauma, these characteristic findings suggest a Purtscher-like retinopathy [1]. 

Purtscher-like retinopathy has been described as being secondary to a myriad of diseases, including pancreatitis, chronic renal failure, and several connective tissue disorders. The rare and varied presentations of this condition have made the development of evidence-based treatment options challenging [1]. Studies in the literature commonly attribute these varied outcomes to the use of steroids and to the strategy of treating only the underlying disease [1-6]. 

Few authors have reported Purtscher-like retinopathy as a complication of systemic lupus erythematosus (SLE). Here, we present the case of a male with unilateral Purtscher-like retinopathy as a complication of SLE. His condition evolved into severe retinal ischemia, retinal neovascularization, vitreous hemorrhage, and ultimately, a combined tractional and rhegmatogenous detachment. 

## Case presentation

A 23-year-old Hispanic male with progressive decreased vision in the left eye (OS) that had endured for three months was referred to the ophthalmology service in the emergency room. The patient’s past medical history was remarkable for SLE, diagnosed four months before his initial visit, for which he had poor follow-up with a rheumatology clinic. At the time of this presentation, he was on prednisone (20 mg oral, twice daily). A review of systems was remarkable for diffuse body pain, headaches, dry mouth, vomiting, nausea, rash, diarrhea, and weight loss, all occurring during the previous four months. His social and family histories were unremarkable. 

**Figure 1:**
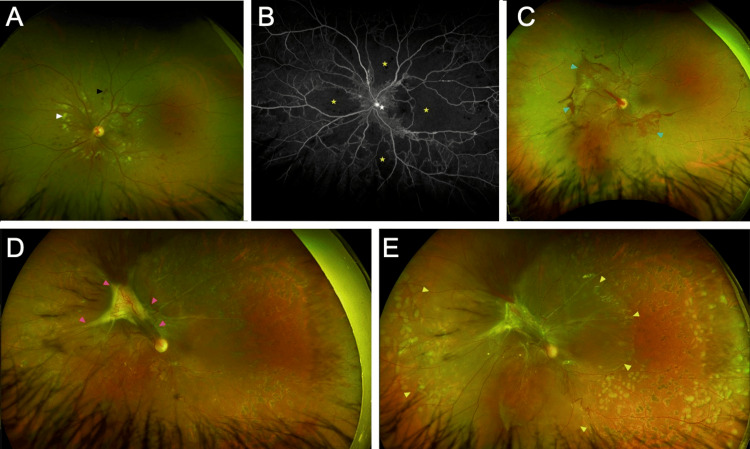
Progressive retinal findings in the left eye over nine months. A color fundus photograph of the left eye (A) at the time of the initial presentation showcases peripapillary cotton-wool spots (indicated by the white arrowhead) and scattered hemorrhages (marked by the black arrowhead). The transit-phase fluorescein angiogram of the same eye (B) displays disc hyperfluorescence, hinting at neovascularization (depicted by the white star), alongside extensive areas of capillary nonperfusion (highlighted by yellow stars). At a five-month follow-up, the color fundus photograph (C) shows sea fan-shaped retinal neovascularization accompanied by vitreous hemorrhages (blue arrowheads). Another follow-up photograph, seven months post-presentation (D), shows a progression to tractional retinal detachment (pink arrowheads); there is also visible evidence of prior scatter laser therapy. At the nine-month mark, a color fundus photograph (E) depicts further evolution to a combined tractional-rhegmatogenous retinal detachment (yellow arrowheads).

Upon a comprehensive ophthalmic evaluation, his best-corrected visual acuity (BCVA) was 20/20 right eye (OD) and counting fingers at 2 feet OS. The intraocular pressure was 18 mmHg OD and 15 mmHg OS (taken with Goldmann applanation tonometry). The pupils were round and reactive to light, and there was an afferent pupillary defect (APD) OS. The APD was most likely due to the severe retinal ischemia. Color vision, as assessed by the Ishihara Color Plate Test, revealed no defect in the OD and was not able to be assessed in the OS. Extraocular movements were within normal limits. A slit-lamp examination was within normal limits, bilaterally, with no evidence of inflammation in the anterior chambers or vitreous cells in either eye. The patient’s fundus was unremarkable in OD; however, the OS revealed peripapillary cotton-wool spots and diffuse hemorrhages (Figure 1A). A fundus fluorescein angiogram depicted transit-phase disc hyperfluorescence, suggesting neovascularization along with extensive areas of capillary nonperfusion (Figure 1B). An assessment of Purtscher-like retinopathy was made, and the patient was admitted for administration of pulse intravenous methylprednisolone therapy (1000 mg) for a period of three days. Brain and orbit magnetic resonance imaging revealed no abnormal findings. Laboratory tests for anti-Smith and anti-ribonucleoprotein antibodies yielded positive results (>480.0 U/mL (normal range: 0-7 U/mL) and >240 U/mL (normal range: 0-20 U/mL), respectively). The tests, which included a complete blood cell count, a comprehensive metabolic panel, a urinalysis, complement C3 and C4 tests, a hepatitis panel, a tuberculosis skin test, a beta 2 glycoprotein test, a centromere B antibody test, a scleroderma antibodies panel, and an aldolase test, were all negative. After completing the pulse of intravenous steroids, he was discharged on prednisone (60 mg oral, daily) and mycophenolate mofetil (1.5 g oral, twice daily). 

At his two-week follow-up visit, his BCVA was 20/20 OD and 20/400 OS. The patient was instructed to gradually taper his prednisone dosage to 10 mg daily. Five months later, his fundus examination showed the presence of sea fan-shaped retinal neovascularization accompanied by vitreous hemorrhages (Figure 1C). Subsequently, he was scheduled for pan-retinal photocoagulation (PRP) laser therapy; after being lost to follow-up, he presented again two months later. A fundus examination revealed the presence of a tractional retinal detachment, and he was immediately treated with PRP (Figure 1D). Ultimately, the patient was scheduled for another round of PRP two months later; however, on that day, he was found to have a BCVA of hand motion OS, and his fundus examination was remarkable for a combined tractional and rhegmatogenous retinal detachment (Figure 1E). He was scheduled for a prompt pars plana vitrectomy. 

## Discussion

Purtscher retinopathy is typically associated with trauma; however, in the absence of trauma, we refer to this clinical entity as Purtscher-like retinopathy (PLR) [1,2]. Pancreatitis, renal disease, and a variety of autoimmune diseases have been reported in various instances of PLR [3,6]. An autoimmune disease primarily impacting young women, SLE, has been linked to the occurrence of PLR [3]. Reports indicate that retinal disease is detected in approximately 3% of those patients with SLE who have well-controlled symptoms, while the prevalence rises to around 29% in those with more active disease [3]. The patient described in this case had recently been diagnosed with SLE, and his disease was not effectively managed at the time of his presentation. 

There are no guidelines for managing PLR, and as patients may recover their vision without any intervention, its prognosis is generally positive. Therefore, some physicians opt for expectant management [2,5]. Nevertheless, the primary focus in treating PLR involves addressing any underlying disease that may be contributing to the retinopathy [5-7]. Although the prognosis tends to be positive, as mentioned above, our case highlights the importance of remaining vigilant, as PLR patients with severe retinal ischemia are at high risk of potentially sight-threatening complications including proliferative retinopathy, tractional retinal detachment, and combined tractional-rhegmatogenous retinal detachment. In such instances, considering treatments such as scattered laser therapy or retinal photocoagulation (with or without adjunctive anti-vascular endothelial growth factor therapy) is crucial. These treatments may not necessarily restore lost vision; however, they do hold the potential to mitigate further complications and preserve the structural integrity of the globe. 

Certainly, PLR may improve spontaneously in some cases [5]. However, in our patient, the severe retinal ischemia rendered this possibility unlikely. Recognizing the potential for a patient to develop sight-threatening complications following severe ischemic retinopathy is crucial. Heightened clinical suspicion may be necessary while managing these circumstances in order to enable early and individually tailored treatment interventions. Further studies should be conducted to compare different treatment strategies and establish standard guidelines. Doing so will not only enhance patient care but also contribute to better outcomes in the presence of this challenging clinical entity. 

## Conclusions

Purtscher-like retinopathy is sometimes seen in conjunction with autoimmune diseases such as SLE. While some PLR cases can spontaneously improve, severe retinal ischemia may lead to significant complications, including retinal proliferation, tractional retinal detachment, and combined tractional-rhegmatogenous retinal detachment. This case underscores the importance of recognizing both the potential for sight-threatening complications in such cases and the need for prompt interventions.

## References

[1] Weng CY. Purtscher Retinopathy and Purtscher-like Retinopathy. EyeWiki (2023). Weng CY. Purtscher retinopathy and Purtscher-like retinopathy. EyeWiki. https://eyewiki.aao.org/Purtscher_Retinopathy_and_Purtscher-like_Retinopathy.

[2] Cheng CK, Lai KK, Kuk AK, Lai TH, Wang ST, Ko ST (2022). Purtscher-like retinopathy in a patient with lupus: a case report. Hong Kong Med J.

[3] Wu C, Dai R, Dong F, Wang Q (2014). Purtscher-like retinopathy in systemic lupus erythematosus. Am J Ophthalmol.

[4] Tripathy K, Patel BC (2022). Purtscher retinopathy. StatPearls [Internet].

[5] Agrawal A, McKibbin MA (2006). Purtscher's and Purtscher-like retinopathies: a review. Surv Ophthalmol.

[6] Miguel AIM, Henriques F, Azevedo LFR, Loureiro AJR, Maberley DAL (2013). Systematic review of Purtscher's and Purtscher-like retinopathies. Eye (Lond).

[7] Gonzalez VH, Wang PW, Ruiz CQ (2021). Panretinal photocoagulation for diabetic retinopathy in the RIDE and RISE trials: not "1 and Done". Ophthalmology.

